# Recovery of brachial plexus lesions resulting from heavy backpack use: A follow-up case series

**DOI:** 10.1186/1471-2474-12-62

**Published:** 2011-03-24

**Authors:** Tuula Nylund, Ville M Mattila, Tapani Salmi, Harri K Pihlajamäki, Jyrki P Mäkelä

**Affiliations:** 1 Centre for Military Medicine, Finnish Defense Forces, (Hennalankatu 259) Lahti (FI-15700), Finland; 2 Department of Clinical Neurophysiology, Helsinki University Central Hospital, (Haartmaninkatu 4) Helsinki (FI-00029 HUS), Finland; 3 BioMag Laboratory, HUSLAB, Helsinki University Central Hospital, (Haartmaninkatu 4) Helsinki (FI-00029 HUS), Finland

**Keywords:** peripheral nerve trauma, peripheral neuropathy, HNPP

## Abstract

**Background:**

Brachial plexus lesions as a consequence of carrying a heavy backpack have been reported, but the typical clinical course and long-term consequences are not clear. Here we evaluated the clinical course and pattern of recovery of backpack palsy (BPP) in a large series of patients.

**Methods:**

Thirty-eight consecutive patients with idiopathic BPP were identified from our population of 193,450 Finnish conscripts by means of computerised register. A physiotherapist provided instructions for proper hand use and rehabilitative exercises at disease onset. The patients were followed up for 2 to 8 years from the diagnosis. We also searched for genetic markers of hereditary neuropathy with pressure palsies. Mann-Whitney U-test was used to analyze continuous data. The Fischer's exact test was used to assess two-way tables.

**Results:**

Eighty percent of the patients recovered totally within 9 months after the onset of weakness. Prolonged symptoms occurred in 15% of the patients, but daily activities were not affected. The weight of the carried load at the symptom onset significantly affected the severity of the muscle strength loss in the physiotherapeutic testing at the follow-up. The initial electromyography did not predict recovery. Genetic testing did not reveal de novo hereditary neuropathy with pressure palsies.

**Conclusions:**

The prognosis of BPP is favorable in the vast majority of cases. Electromyography is useful for diagnosis. To prevent brachial plexus lesions, backpack loads greater than 40 kg should be avoided.

## Background

Brachial plexus lesions in association with carrying a heavy backpack (backpack palsy, BPP) have been reported in soldiers [1-4], boy scouts [5], and in association with sports such as hiking [6] and mountaineering [7], or with manual labor (e.g., carrying sandbags) [8]. BPP typically presents with paresis, numbness, and paresthesias of the upper extremity after carrying a heavy backpack. The painless motor weakness most severely affects the shoulder girdle and elbow flexors. Sensory disturbances occasionally occur in the lateral shoulder and arm region and in the radial aspects of the forearm and hand. Transient weakness of the upper limb may precede the full syndrome. A distinct atrophy of the affected muscles sometimes develops during follow-up [1-4,7]. Carrying heavy backpacks without waist support, particularly when working with the hands in difficult terrain, managing a particularly heavy load, and long exposure duration increase the risk of BPP [1,2]. Poorly developed shoulder musculature [5] and prior anomalies or injuries to the shoulder or vertebral regions [1] may predispose to BPP. Good physical fitness does not decrease the risk of BPP [4].

Sporadic or hereditary neuralgic amyotrophies, probably autoimmune in origin, present with very severe neuralgic pain in association with brachial plexus paresis and sensory findings [9-11]. BPP is easily distinguished from these clinical entities, however, as it is generally painless and is not usually associated with sensory abnormalities. The prognosis of neuralgic amyotrophies has been described in detail, but patients with possible pressure palsies or traction of the brachial plexus were excluded from these follow-ups [9,11]. The pattern of recovery from BPP, therefore, is not clear. Two-thirds of patients recover fully or almost fully within 2 [1] to 5 [2] months; the clinical course of the other one-third of patients with a prolonged recovery is not known.

The long thoracic nerve, which is often affected in BPP, recovers on average within a mean of 9 months and as late as 2 years after injury [12]. Symptoms of iatrogenic serratus anterior paralysis, however, may persist for well over 6 years after long thoracic nerve injury [13]. Patients are often young males with poorly defined vocational and educational patterns, or those dependent on manual skills, and a potential handicap resulting from upper arm dysfunction can be particularly harmful to their professions and daily living activities. Therefore, a detailed prognosis would be useful to evaluate the inconvenience caused by the lesion and to aid in educating the patient about possible future consequences [14]. We followed a large series of patients with BPP [4] to determine the natural history and consequences of BPP lesions in more detail.

Hereditary neuropathy with pressure palsies (HNPP) increases the risk for BPP [15]. This autosomal dominant disease results from a deletion on chromosome 17p11.2-12, which includes the peripheral myelin protein 22 gene. Approximately 25% of the deletion carriers are symptomless. In one study of a small series of patients, all of the patients with brachial plexus lesions carried the gene deletion typical for HNPP [15]. Brachial plexus involvement may be the sole manifestation of HNPP [16]. We searched for a genetic marker of HNPP in all examined patients to determine if HNPP without clinical or electromyography (EMG) markers causes BPP.

## Methods

### Participants

**Figure 1:**
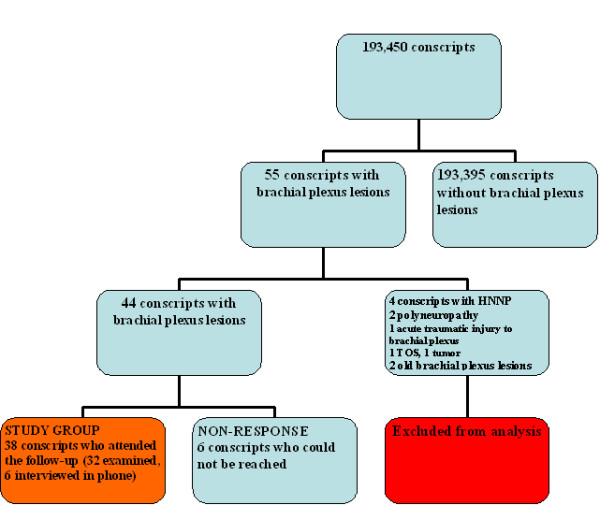
**Brachial plexus lesions among 193,450 military recruits and selection of study participants**.

Consecutive military recruits with symptoms of BPP after carrying a heavy load on the shoulders were identified from the medical records of the Central Military Hospital from the years 1998--2004 using ICD-10 diagnosis codes for brachial plexus compression, and lesions of the long thoracic, suprascapular, axillary, musculocutaneous, median, radial, and ulnar nerves. In addition, all EMG reports from this period were reviewed to confirm that all patients with BPP were identified. A total of 55 (1 female) patients with BPP were treated from 1998 to 2004. The incidence was 53.7 per 100 000 military conscripts per year [4]. To limit the follow-up study to patients with truly idiopathic BPP, 11 patients were excluded (Figure 1). Written informed consent was obtained from each patient. The Medical Ethics Committee of Helsinki University Central Hospital approved the study design.

### Interventions

All patients with BPP underwent a physical examination and EMG at the symptom onset during military service [4]. After confirming the diagnosis, the physiotherapist provided instructions for proper hand use and appropriate exercises to maintain shoulder function. Military duties were limited to tasks not requiring the use of the affected arm. If the symptoms continued at the end of the military service, the patients were referred to the general health care system

### Follow-up

Follow-up time begun at the time of the diagnosis during the military service and ended at the follow-up examination after the military service. The median follow-up time was 4.5 years (range, 2 - 8 years), 38 of the 44 patients participated the follow-up.

### Tests

The patients completed a structured questionnaire about the circumstances at symptom onset and about the condition of the upper limb. The weight estimate of the load was specifically questioned from each subject and compared with data in the initial reports. Moreover, particular effort was put to find out about the type of the carried load at the onset of symptoms. As we know the weight of different types of military equipment, we could then produce an estimate about the carried load (Table 1).

**Table 1:** Follow-up characteristics of the 38 patients with PBB.

Follow-up characteristics	Yes	No	
Self-reported symptoms at the follow-up	8/38 (21%)	30/38 (79%)	
Normal muscle strength*	27/32 (84%)	5/32 (16%)	
The lesion affected the patient's profession after the army	5/38 (13%)	33/38 (87%)	
Decreased physical activity due to the lesion after the army	5/38 (13%)	33/38 (87%)	
	Muscle strength at the follow-up*		
	Normal	abnormal	
The weight of the load carried (mean, SD)	30 kg (10 kg)	44 kg (6 kg)	p = 0.003**
Number of affected nerves at the baseline			
1	17 (81%)	4 (19%)	p = 0.999***
2	6 (75%)	2 (25%)	
3	3 (100%)	0 (0%)	
most affected peripheral nerve			
long thoracic	9 (75%)	3 (25%)	p = 0.847***
Musculocutaneus	2 (100%)	0 (0%)	
Axillary	10 (83%)	2 (17%)	
Median	1 (100%)	0 (0%)	
Suprascapular	3 (100%)	0 (0%)	
Radial	1 (100%)	0 (0%)	
Ulnar			
	Self-reported symptoms at the follow-up		
	No	Yes	
The weight of the load carried (mean, SD)	38 kg (6 kg)	42 kg (7 kg)	p = 0.249**
Body mass index (mean, SD)	22.1 (3.8)	23.6 (4.1)	p = 0.518**

* Only for those attending the follow-up examination (n = 32)

** Mann-Whitney U-test

*** Fischer's exact test

Thirty-two patients completed the questionnaire, clinical neurological assessment, physiotherapeutic muscle tests, EMG, and genomic DNA isolation from the peripheral blood. DNA was digested by restriction enzymes using a Nucleon BACC3 kit (GE Healthcare). The copy number of the peripheral myelin protein 22 gene was estimated by the presence or absence of the deletion junction fragment, and visually from polymorphic bands on autoradiograms [17,18]. In addition, six patients completed the questionnaire by phone (Table 1). Results of clinical tests or blood samples were not available from these patients.

The main outcome variable was self-reported symptoms (yes/no) in the anatomic location affected by BPP. Additional outcome variables were also assessed. Muscle strength, recorded by a physiotherapist (range 1 to 5, compared with the unaffected side), was re-coded to a dichotomous variable (normal/abnormal muscle strength). The length of the total recovery period in months was determined. The level of physical activity at the time of the examination was scored (range, 1 [none] to 5 [more than 4 times per week]).

Findings in EMG were compared with those obtained at disease onset [4]. EMG and conduction velocity studies were performed using a Keypoint^® ^EMG device (Medcare, Buffalo, NY). The motor responses and distal latencies of the affected proximal and distal nerves were recorded using surface electrodes and compared with the corresponding contralateral site. In needle EMG, fibrillation due to denervation was evaluated in the muscles that were affected in the baseline EMG study. Reinnervation was evaluated by visual analysis of the activating motor unit potentials. Single-fiber EMG analysis was applied to determine the increased fiber density due to reinnervation [19,20]. The EMG findings were rated as normal (0), slightly abnormal (1), significantly abnormal with weakness and some paresis (2), and marked paresis (3).

### Background variables

We recorded background variables to evaluate their association with recovery. The patients were interviewed, and the primary care patient records were examined. Height and weight were obtained from the original medical records and body mass index (BMI) was calculated as weight (kg)/(height [m])^2^.

### Data analyses

The associations between the self-reported symptoms, muscle strength, mean load carried at the time the symptoms began, number of affected nerves, BMI, and body structure were evaluated. The patients (n = 6) not attending the clinical examination at the follow-up were interviewed by phone and included into analysis of self-reported symptoms and self-reported muscle strength. They were excluded from the other analyses (Table 1). We also investigated the association between recovery as measured by EMG and clinical status and the affected nerve (long thoracic, suprascapular, axillary, musculocutaneous, median, radial, and ulnar nerve). Mann-Whitney U-test was used to analyze continuous data. The Fisher's exact test was used to assess two-way tables. The differences between two independent groups were considered statistically significant if the p value was 0.05 or less. SPSS 14.0 for Windows (SPSS, Chicago, IL) was used for statistical analysis.

## Results

### Recovery

After a median follow-up of 4.5 years, 30 of the 38 patients (79%; 95% CI: 64-90%) reported that they were asymptomatic. Of the 32 patients that attended the follow-up examination, 27 (84%) had normal muscle strength. Strength overcame gravity in all five of the affected patients. Decreased muscle strength, however, was not associated with self-reported symptoms (p = 0.198).

In patients with total recovery, the median time from symptom onset to total recovery was 3.2 months (range 0-9 months). The lesion affected military duties in seven cases, leading to a change in service qualification classification. The lesion affected the patient's profession after the army in five cases, and five patients had decreased their physical activity due to the lesion. The patients with symptoms at the follow-up had a median follow-up time of 4 years (range, 2 - 5 years) at the time of testing.

### Predictors of symptoms

The estimated weight of the load carried at the time of symptom onset was associated with muscle strength at the follow-up, and a more severe injury was observed in those who had carried a heavier load (on average, 30 kg in those with normal muscle strength at the follow-up and 44 kg in those with abnormal muscle strength at follow-up; p = 0.003; Table 1). No significant association was detected, however, between symptoms at follow-up and carried load (p = 0.249). Nine patients reported that they continued to carry a heavy load after the initial symptoms, and recovery was not affected. BMI (p = 0.518) was not associated with the self-reported symptoms at follow-up. The number of affected nerves (range 1 to 3) at baseline was not associated with the self-reported symptoms (p = 0.218) or muscle strength at follow-up (p = 0.999).

### Predictors of EMG results

The most affected peripheral nerve of the arm, as diagnosed using the first EMG, was not associated with the symptoms (p = 0.120) or muscle strength (p = 0.847) at follow-up (Table 1). The EMG score at follow-up was not associated with the symptoms (p = 0.435) or muscle strength (p = 0.401). No positive HNPP gene tests were detected in any of the studied patients.

## Discussion

In the present study, 80% of the patients recovered from BPP within a median of three months (range 0-9 months) of incurring the lesion. Although 15% of the patients had prolonged symptoms, daily activities were not affected. Five patients reported some difficulties in their present profession and five had modified their free-time physical activities because of the symptoms. One study reported that brachial plexus injury leads to absence from the job of more than 2 years in 44% of affected patients [21], and persistent pain and paresis occurs in approximately two-thirds of neuralgic amyotrophy patients after a follow-up of 3 years [11]. In our study, BPP had no such dire consequences, and the prognosis was favorable in the vast majority of the cases.

The weight of the carried load at the onset of symptoms, estimated at follow-up, was significantly related to the severity of the muscle strength loss in the physiotherapeutic testing at follow-up; however, this finding was not generalized to subjective symptoms. Weights exceeding 40 kg should be avoided in long-term carrying to avoid such chronic effects.

BMI did not predict recovery in our study; nor did it predict the occurrence of the lesion in our previous study [4]. The patients' grades in gymnastics were high, and some were athletes competing at the national level. Consequently, the lesion and its recovery are difficult to predict based on such factors. EMG results at the onset of the lesion were useful for detecting lesions, but not for predicting recovery.

The prevalence of HNPP in southwestern Finland is at least 16/100 000 [18], which is within the same order of magnitude as that observed for brachial plexus involvement in military conscripts (54/100 000) [4]. We previously searched for HNPP on the basis of clinical and EMG findings [4]; the incidence was 4/100 000, less than that indicated by surveys of the general population [18]. If EMG findings were limited to the "typical" area of the brachial plexus in BPP, no new HNPP patients were found, consistent with a previously described high concordance between the EMG findings and the DNA analysis [18]. The lower prevalence of HNPP in our study than in the general population is probably due to pre-service screening; in Finland, patients with known HNPP are released from military service.

Several patients continued carrying their backpacks despite clear symptoms. In some cases, the patients had complained about the symptoms but were persuaded to continue carrying the backpacks by their superiors, consistent with reported experiences elsewhere [21]. Prolonged symptoms might be prevented or diminished by an increased awareness of the condition.

## Conclusions

Our findings indicated that 80% of the patients recovered completely from BPP within 9 months after the injury, and no problematic hand or arm dysfunction was detected in the remaining patients. EMG at the subacute phase is useful for diagnosis, but does not predict recovery. Routine genetic testing for HNPP, at least by the method we applied, in patients with typical BPP is not warranted. To prevent brachial plexus lesions, backpack loads greater than 40 kg should be avoided.

## Abbreviations

BMI: body mass index; BPP: backpack palsy; EMG: electromyography; HNPP: hereditary neuropathy with pressure palsies; ICD: international classification of diseases.

## Competing interests

The authors declare that they have no competing interests.

## Authors' contributions

TN and JM collected the patients. TS analyzed the ENMG recordings. VM participated in the design of the study and performed the statistical analysis. TN, JM TS, VM and HP conceived of the study, and participated in its design and coordination and helped to draft the manuscript. All authors read and approved the final manuscript.

## Pre-publication history

The pre-publication history for this paper can be accessed here:

http://www.biomedcentral.com/1471-2474/12/62/prepub
